# A catalog of metagenome-assembled genomes from Amazonian forest and pasture soils

**DOI:** 10.1128/mra.00642-25

**Published:** 2025-10-02

**Authors:** Andressa M. Venturini, Júlia B. Gontijo, Louis Berrios, Jorge L. Mazza Rodrigues, Kabir G. Peay, Siu M. Tsai

**Affiliations:** 1 Department of Biology, Stanford University 6429, Stanford, California, USA; 2 Department of Environmental Science, American University 8363, Washington, DC, USA; 3 Department of Land, Air, and Water Resources, University of California Davis 8789, Davis, California, USA; 4 Department of Earth System Science, Stanford University 6429, Stanford, California, USA; 5 Cell and Molecular Biology Laboratory, Center for Nuclear Energy in Agriculture, University of São Paulo 28133, Piracicaba, São Paulo, Brazil; DOE Joint Genome Institute, Berkeley, California, USA

**Keywords:** Amazon rainforest, deforestation, land-use change, soil microbiology, bacteria, archaea, metagenomics, antimicrobial resistance genes, virulence genes

## Abstract

The Amazon rainforest is facing multifaceted anthropogenic pressures, and we previously showed that forest-to-pasture conversion has led to soil microbial communities with distinct genomic traits. Here, we present 69 archaeal and bacterial metagenome-assembled genomes and detail their virulence- and antimicrobial resistance-associated genes.

## ANNOUNCEMENT

Our group previously recovered 64 bacterial and 5 archaeal metagenome-assembled genomes (MAGs) from Amazonian forest and pasture soils and evaluated their genomic traits (1). Here, we formally announce these genomes and provide additional characterization by reporting the presence of plasmids, virulence-associated genes, and antimicrobial resistance genes (ARGs). Changes in the resistome, including an increase in the abundance of ARGs, have been previously detected in association with forest-to-pasture conversion in the Amazon (2, 3).

Sampling was carried out in three forests within the Tapajós National Forest and three cattle pastures nearby, in the state of Pará, Eastern Amazon, Brazil, in November 2015 and May 2016. Three samples (0 to 10 cm depth), spaced 100 m apart, were collected at each site in both sampling periods using polyvinyl chloride tubes. Total soil DNA was extracted in duplicate using the PowerLyzer PowerSoil DNA Isolation Kit (QIAGEN GmbH, Hilden, Germany), following the protocol by (4). DNA samples were evaluated and subjected to shotgun metagenomic sequencing (2 × 150  bp) on an Illumina HiSeq (Illumina, Inc., San Diego, CA, USA) at Novogene Co., Ltd. (Beijing, China), with libraries prepared using the NEBNext Ultra II DNA Library Prep Kit for Illumina (New England Biolabs, Inc., Ipswich, MA, USA). Comprehensive details on the sampling procedures, molecular and bioinformatics methods, and metagenomic sequences can be found in the Supplemental material of (1), including both the text section and Tables S1 and S2.

**Fig 1:**
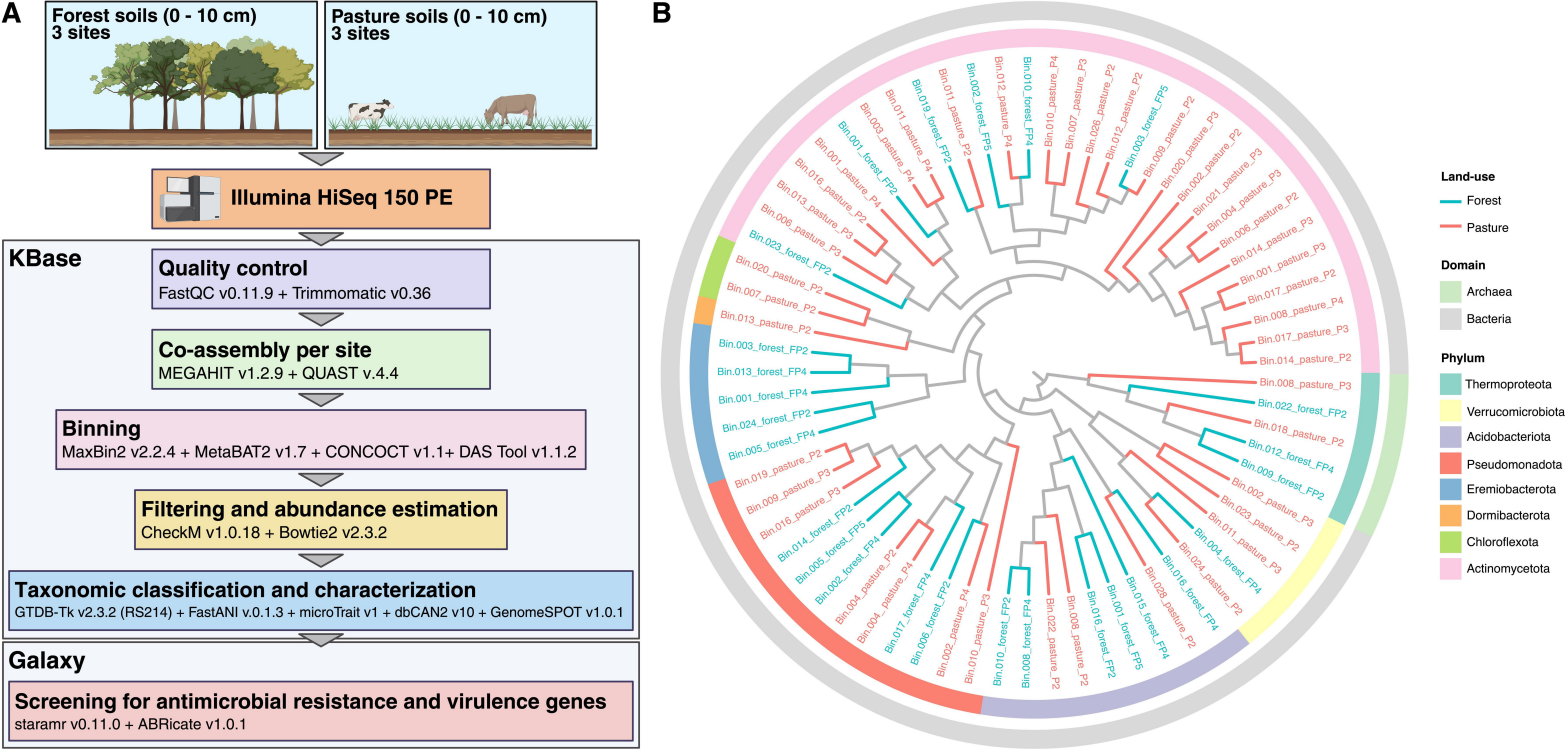
(**A**) Overview of methods and software used for the recovery and characterization of MAGs from forest and pasture soils in the Brazilian Amazon. For each site, soil metagenomes were merged and co-assembled. MAGs were recovered using binning tools based on contig sequence composition and coverage from the merged data sets and then characterized and taxonomically classified (5, 7, 8, 12, 15–31). The figure was created with BioRender (Venturini, A. M., 2025, https://BioRender.com/srdoltg). (**B**) Phylogenomic tree of the 69 MAGs recovered. The outer ring indicates the domain of each MAG, and the inner ring represents the phylum. Branches are colored by land-use type.

Sequences were processed on the KBase platform (5), where they were filtered, trimmed, and co-assembled into contigs by site. Contigs were binned, filtered, classified taxonomically, and further characterized, as described in Fig. 1A and (1) (Supplemental material). Medium- and high-quality MAGs were used as inputs for phylogenomic analysis using a set of 49 universally conserved genes from Clusters of Orthologous Groups gene families in the Build Microbial SpeciesTree 1.6.0 app (5), and the resulting tree was visualized using TreeViewer v. 2.2.0 (6). MAGs were imported into the Galaxy platform v. 24.2 (7), where screening for antimicrobial resistance and virulence genes was performed using ABRicate v1.0.1 (8), with the Virulence Factor Database (9), the NCBI Bacterial Antimicrobial Resistance Reference Gene Database (10), and the Comprehensive Antibiotic Resistance Database (CARD) (11), as well as staramr v.0.11.0 (12), with ResFinder (13) and PlasmidFinder (14), using default parameters.

We recovered 69 archaeal and bacterial MAGs (26 from forests and 43 from pastures), spanning 8 phyla (Fig. 1B), which exhibited distinct genomic and functional traits (1). MAG statistics, taxonomic classifications, and functional annotations are provided in (1) (Supplemental material, Tables S4–10), and a summary of all data sets, including accession numbers, is also available on Figshare (doi.org/10.6084/m9.figshare.29998138). No plasmids or acquired ARGs were identified using staramr (12). However, ABRicate v1.0.1 (8) detected virulence genes and ARGs in pasture MAGs. Specifically, 11 virulence-associated genes were found in a *Mycobacterium* MAG (genus of the phylum Actinomycetota; Bin.014_pasture_P2; *eccA3*, *eccA5*, *eccCb5*, *esxH*, *fbpA*, *fbpB*, *hbhA*, *icl*, *mbtG*, *mbtH*, and *phoP*) and one virulence gene was detected in two Xanthobacteraceae MAGs (family of Pseudomonadota; Bin.019_pasture_P2, Bin.009_pasture_P3; *acpXL*). All hits had ≥ 88% of coverage and ≥ 80% of nucleotide identity. In addition, *vanR-O* genes (associated with vancomycin resistance) were found in eight pasture MAGs (Bin.028_pasture_P2, Bin.001_pasture_P3, Bin.007_pasture_P3, Bin.020_pasture_P3, Bin.021_pasture_P3, Bin.008_pasture_P4, Bin.010_pasture_P4, and Bin.011_pasture_P4), seven of which belonged to Actinomycetota. These genes showed coverage ≥ 93% and nucleotide identity ≥ 83%. Additional antibiotic resistance and resistance-associated regulatory genes were identified in three Actinomycetota MAGs (*efpA*, *mtrA*, and *RbpA* in Bin.014_pasture_P2; *rpoB2* in Bin.017_pasture_P3; and *vanSO* in Bin.021_pasture_P3; all with ≥ 83% of coverage and ≥ 81% of nucleotide identity).

## Data Availability

The raw, cleaned/filtered, and merged metagenomic sequences, along with the co-assemblies and metagenome-assembled genome (MAG) sequences, are available on the KBase platform at https://doi.org/10.25982/159671.262/2496635. The raw metagenomic sequences and MAGs are also available at NCBI under the umbrella project PRJNA1112097. A table containing metadata, summary statistics, and NCBI accession numbers for the metagenomes, co-assemblies, and recovered MAGs is available on Figshare (https://doi.org/10.6084/m9.figshare.29998138).
